# Priorities in effective management of primary health care institutions in Lithuania: Perspectives of managers of public and private primary health care institutions

**DOI:** 10.1371/journal.pone.0209816

**Published:** 2018-12-31

**Authors:** Aida Budrevičiūtė, Ramunė Kalėdienė, Jadvyga Petrauskienė

**Affiliations:** Department of Health Management, Faculty of Public Health, Medical Academy, Lithuanian University of Health Sciences, Kaunas, Lithuania; National University of Ireland Galway, IRELAND

## Abstract

**Background:**

Primary health care institutions are looking for opportunities to create value for patients and to increase the competitiveness of the health care institution. Determination of competitive priorities for creation of value for patients in the management of primary health care institutions allows improving competitiveness and achieving a competitive advantage in the market.

**The aim of the study:**

To determine the priorities in the management of public and private primary health care institutions by using the focus group discussion method with managers.

**Methods:**

The study was exploratory with intention to find a ground for a management theory and to be the root for the development of health care reform in Lithuania. Focus group discussions were held in 10 Lithuanian counties; 10 focus group sessions were carried out. A total of 48 primary health care executives were interviewed. The participants of this qualitative study were given 8 questions related to value creation of the primary health care institution to patients and rise in competitiveness. The main question of the focus group discussion was “What are the main priorities of management of primary health care institution?” The criteria of data collection based on the deep understanding of the phenomenon and the richness of data expressed by participants of the research.

**Results:**

Qualitative research showed that the priorities of management of primary health care institutions were work management of an organization; human resources management; patient management; and health policy decision making. The participants of focus groups pointed out that effective work of primary health care institutions is ensured by the model of management, doctor-patient communication, quality and timely delivery of health care services, and financial resources. The major decisions involving the management of patients were as follows: meeting patients’ expectations, quality and timely satisfaction of patients’ needs, effective solution of patients’ problems, patient-centered services, patient satisfaction, and communication with the patient. Accessibility to services, quality, geographical accessibility, disease prevention, strengthening of patients’ health and adequate funding were mentioned as the priorities of health policy.

## Introduction

Primary health care institutions focus on the priorities of modern management how to create value for patients. In the United States (US), the development approach of primary health care relationship-centered practice that includes people and places where the functions of access to first-contact care, comprehensive care, coordination of care, personal relationship over time are improved has been proposed [1]. Primary health care institutions are becoming high-performing patient-centered organizations providing the key elements of primary care, building blocks that include foundational elements such as engaged leadership, data-driven improvement, empanelment, and team-based care, assisting the implementation of other building blocks such as patient-team partnership, population management, continuity of care, prompt access to care, comprehensiveness and care coordination, and a template of the future [2]. Decisions about priority setting can be made at different levels, including international, national, regional, organizational, and employee- and patient-centered. In Sweden, there are three main criteria employed by health care authorities to develop national guidelines for priority setting: severity of health condition, expected patient benefit, and cost-effectiveness of medical intervention [3]. In the study that used focus groups for data collection, additional dimensions of priority setting in primary health care–medical or patient’s viewpoint, timeframe, and group or individual evidence level–were identified [3]. The results of quantitative research on patients (n = 2517) in primary health care in Sweden showed that 22% of the patients disapproved a definite position on priority setting and 6% of the patients expressed a positive attitude to prioritizing [4]. The consultation with a family doctor is fundamental to the delivery of primary health care services. In the UK, patients (n = 1193) from family practices were surveyed about their priorities in primary health care, and this study showed that patients might give a higher priority on the technical quality of care and continuity of care [5]. Priority setting in primary health care is an open space for research with different scientific methods. The sociocultural context of the study described by health inequalities in Lithuania that are exceptionally high according to gender, age, education, income, residence, access to health care [6]. It was described that groups of population with lower education, manual occupations, unemployed, economically inactive, unmarried had the most unfavourable position in terms of mortality in Lithuania [6].

### Health care reform in Lithuania and development of objectives of the research

Before the health care reform in Lithuania, provision of the services provided by specialist doctors predominated. In 1991, the Supreme Council of the Republic of Lithuania approved the National Health care Concept, which aims to restructure health care services and to focus on the primary health care and family health care institution. It is argued that the development of a primary medicine institution is a priority in management [7]. The main objectives of the first phase (2003–2005 year) were to improve health care quality and accessibility to services, to optimize the scope and structure of health care needs. In order to improve the quality of health care services and accessibility, the strategy of health care restructuring was approved. Restructuring of health care is carried out in three priority areas: outpatient services, especially development of primary health care institutions; optimization of inpatient services and alternative forms of activity; and medical care and long-term nursing services [8]. The second phase (2006–2008 year) of health care system restructuring involves the separation of primary and secondary level outpatient services with an emphasis on primary health care organization in rural areas and development of the network of private primary health care institutions [9]. The second phase of health care restructuring was designed to optimize the health care network and improve the service structure, based on health care needs in the population [10]. In the second phase, one of the key objectives was to develop a patient-centered primary health care institution and to achieve that by the end of 2008 at least 60% of primary health care services would be provided by privately employed family doctors [10]. The third stage (2009–2011 year) of the restructuring of health care institutions and services aimed to provide safe, high-quality, and accessible health care services to the population and efficient use of health care resources [11]. The aim was to ensure that primary health care would cover person-oriented, primary dental and primary mental health care, and the most rational use of human, material, financial resources. One of the main objectives was the development of private primary health care institution activities [11]. The fourth phase (2012–2016 year) of health care system development and hospital network consolidation regarded the development of the main direction outpatient services, particularly primary health care and disease prevention strengthening [12]. The importance of the primary health care system is defined by service provision to patients while focusing on meeting the patients’ expectations and needs and improving patient satisfaction. Increasing competition among primary health care institutions gives patients the freedom to choose a health care institution and a doctor. Meeting the patients’ expectations with health care services, primary health care human resources, and patients’ partnership are the core priorities in the management of primary health care institutions. This qualitative study is important for the assessment of health care reform progress in Lithuania and to be the strategic direction for the health care reform development. The value of this study is defined by the chosen research method, executives of primary health care institutions were interviewed by using focus group discussions. The following objectives of the research were formulated:

1^st^ objective. To determine the priorities of competitive advantage of the primary health care institution.
2^nd^ objective. To explore a key source of competitive advantage of the primary health care institution.
3^rd^ objective. To describe essential activities of patient management of the primary health care institution.
4^th^ objective. To reveal decisions that increase the competitiveness of the primary health care institution.

### Ethical aspects

Scientists addressed the ethical aspects in qualitative research that combine relationships between researchers and participants, consent and anonymity, privacy and confidentiality [13]. In qualitative studies participant’s world include participant recruitment, representations of participant’s voices, involvement of vulnerable populations and ethical challenges related to the researcher’s world include risk management, the process of emotional content [14]. The methodology of qualitative research is driven by theoretical assumptions and by choice of techniques [15]. The qualitative research conducted by awareness of the process and well-established ethical principles: autonomy (participants exercise their rights as autonomous persons), beneficence (research is doing good for others), justice (equal share and fairness) [16]. The permission (No. BE-2-11) to conduct the research was given by Kaunas Regional Committee of Biomedical Research Ethics (Lithuania). In the permission were discussed ethical aspects about participants expectations about their role in the research, representation aims and values of the study, participant role and identity. The informed consent to participate in the qualitative research approved by Kaunas Regional Committee of Biomedical Research Ethics (No. BE-2-11). The authors applied to the Lithuania State Data Protection Inspectorate for study’s participant personal data protection (No. DVT2-2009).

## Material and methods

**Fig 1:**
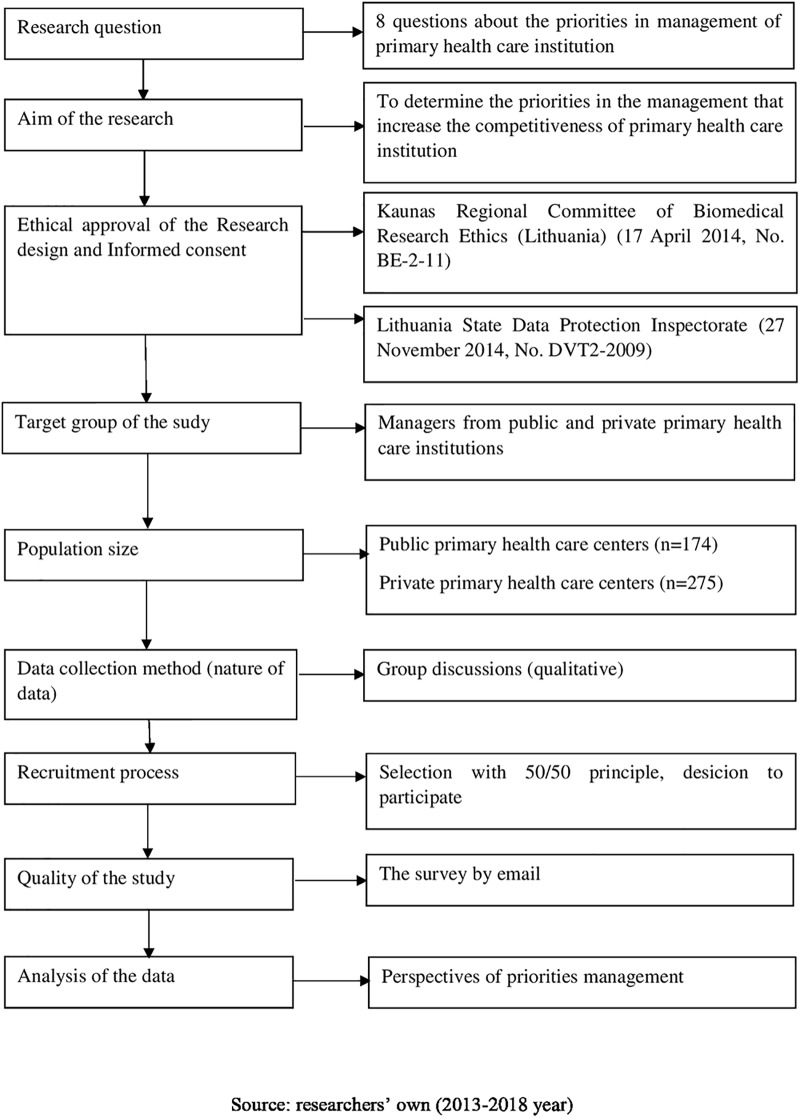
The methodology of the study.

The qualitative research is an interactive, iterative process of participants and researchers jointly exploring the phenomenon to yield rich insights for theory building [17, 18]. Data gathered through focus group sessions by sharing ideas, attitudes, insights, and experience on the chosen topic among the participants [17]. The deepest level of understanding provided by judgments, assumptions, behaviors, intentions [18]. In Lithuania primary health care institutions differ by ownership form, patients could choice the primary health care institution and there working the family doctor who could work in public or private primary health care institution. The methodology of the study is presented in the Fig 1.

The mean size of the focus group was 5 participants. The mean duration of the focus group discussion was 1.21 h. The focus groups study was carried out in this sequence:

- Participants for this focus group study were selected from the list composed by the Lithuanian Institute of Hygiene at the end of 2012. Selection was done following the principle of 50/50, with the intention to include the executives of both public and private primary health care institutions.
- The executive of the primary health care institution was contacted by phone and informed about the topic, purpose, time and place of group discussions.
- As soon as the leader of the primary health care institution agreed to participate in the focus group session, an invitation was sent by e-mail and/or presented to the participant or to hospital staff, which gave the invitation to the executive.
- After informing about focus group discussions and agreement to participate, informed consent to participate in focus group sessions was obtained.
- Focus group sessions were audiotaped and based on the records, analysis of focus group discussions was performed.
- In order to assess the quality of focus group discussions, the questionnaires were sent to participants of focus group discussions by e-mail after the study.

The focus group discussions involving the executives of primary health care institutions were conducted from May 2015 to March 2016 in 10 counties of Lithuania: Vilnius, Kaunas, Klaipėda, Šiauliai, Panevėžys, Utena, Alytus, Marijampolė, Tauragė, Telšiai. A total of 48 participants were enrolled into the qualitative study: 31 leaders of public primary health care institutions and 17 leaders of private primary health care organizations. The profile of participants is described in the Table 1.

**Table 1:** Profile of participants.

Gender	Executives from private primary health care institution	Executives from public primary health care institution
Female	11	20
Male	6	11
Total	17	31

**Fig 2:**
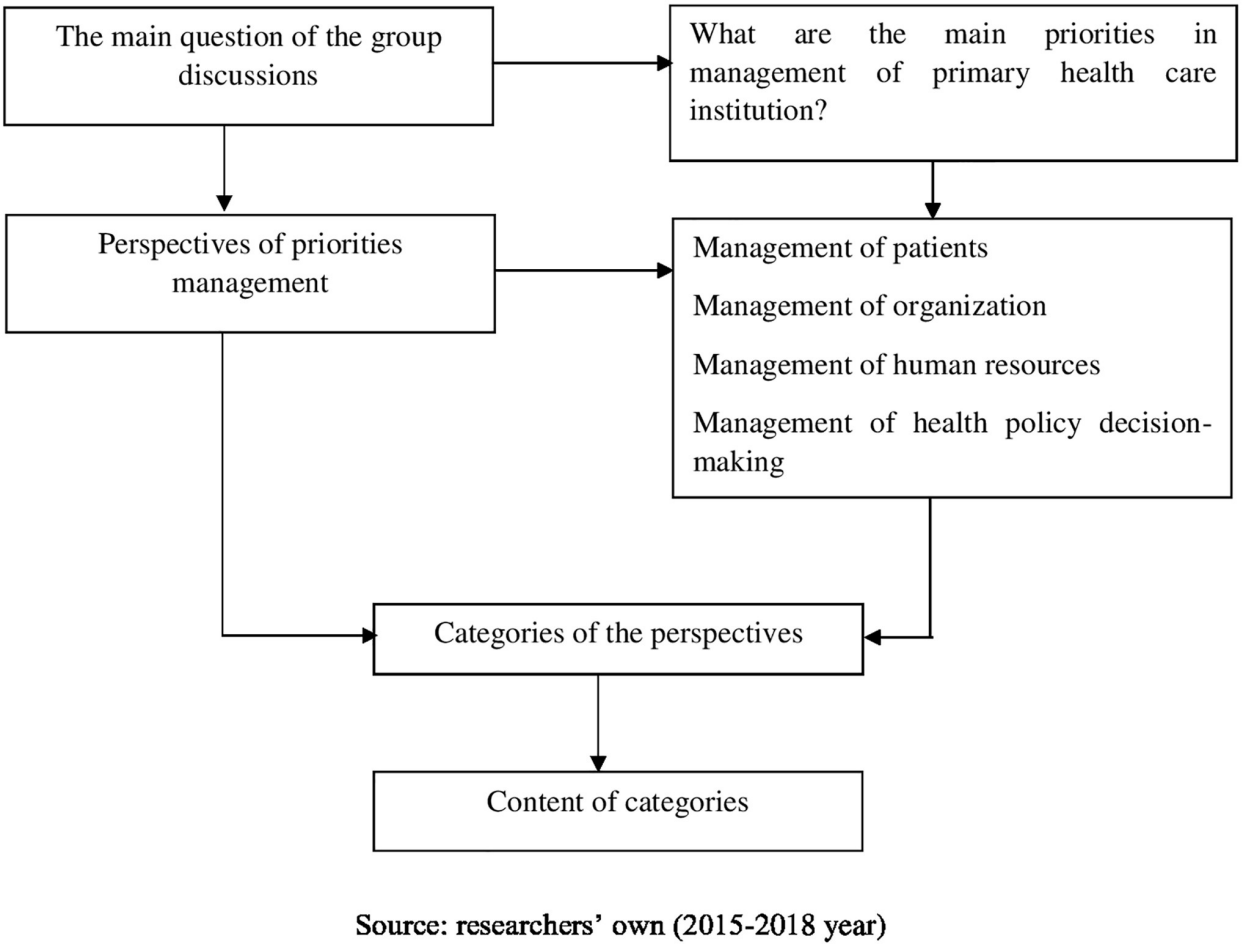
Proposed methodology of data analysis.

Qualitative study analysis revealed the opinion of the participants of focus group discussions toward management priorities of the primary health care institution. The study analysis was done based on participants’ opinion, insights, and experience. The analytical approach of the qualitative study was based on the Grounded theory and the researchers following the principles of the rigor and quality of the analysis (Fig 2).

The data were analyzed as follows:

1. The data were categorized into priorities of “Management of organization,” “Management of human resources,” “Management of patients,” “Management of health policy decision-making” from the participant perspective.
2. Each category was defined and differences and similarities were established. If the analysis categories related to the perspectives, it was predefined by the research team.
3. The data of the qualitative study were grouped by perspectives, categories and explained its content.

## Results

The results of this qualitative study showed that priorities in the management of primary health care institutions were related to organization management, human resources management, patient management, and of health policy decision making.

### Management of organization

The study showed that “management of organization” perspective was divided into the following categories by the executives of public primary health care institutions: concept of family physician specialty, accessibility, examination, workload management, communication, quality, financial resources, patients, health promotion activities, image building, and decision management (Table 2).

**Table 2:** Categories of “management of organization” perspective.

Public primary health care institutions		Private primary health care institutions	
Categories	Content of category	Categories	Content of category
Family doctor	A family doctor has no time to deal with social issues At least 1–2 times a year, the patient should visit a family doctor	Management of services	Rate of service provision Timely provision of services Time of services Provision of services by phone Time management of services
Accessibility	Registration Access to family doctors Calls to the patient’s home Management of revisits Patients’ queue management	Accessibility	Patients’ queue management
Examination	Examination performance at the primary level, and payment for additional medical services at the primary level	Principles of marketing	Place of health care organization Priority in patients number of growth
Management of workload	Necessary to carry out a number of services Workload of family doctor and management (work volume, financial resources) Provision of health care services to patients Services at the secondary level are provided in the same building as those at the primary level Occupational safety Working procedures Document management procedures Accreditation is business card (procedures, management, and other managerial procedures) Infrastructure	Management of workload	A family doctor solves 80% of patient’s problems Occupational safety
Communication	Doctor-patient communication Suitable provision of health care services	Communication	Contact of family doctor-patient Culture of staff communication Inter-institutional communication Relations with partners (school workers, hospital staff, social workers)
Quality	Quality of services	Quality	Quality of service Quality is the organization’s activities corresponding to a contract with the health insurance funds and medical rate
Financial resources	Financial resources of organization	Financial resources	Attraction of additional financial resources
Patients	Number of patients Retention of existing patients	Management of documents	Documents
Health promotion	Promoting healthy lifestyles Implementation of health programs Health coordinators engaged in health programs and communication with a patient	Health promotion	Diseases prevention Projects of health promotion
Management of decisions	Management model of organization	Management of decisions	To provide assistance to people at the specified level Effective organization (administrative priorities)
Image building	The role of media in shaping a positive image of health care organizations, information about health programs		

The participants of private primary health care institutions divided “Management of Organization” category into workload management, accessibility, principles of marketing, services management, management of documents, communication, quality, financial resources, health promotion activities, and management of decisions. Each primary health care institution has its own management model, which includes the organizational structure, financial resources, human resources management, patient management, quality systems, and infrastructure:

…*The institution has to have its own management model and the priority is a patient followed by accessibility*, *quality*, *etc*.

(woman, public primary health care, Panevėžys county)

According to the opinion of executives from public primary health care institutions, the family doctor should not address patients’ social problems, but should provide medical services. One of the most important priorities of management is family doctor-patient communication by ensuring adequate health care services to the patient. The executives of public primary health care institutions reported that the number of patients registered with an institution and their retention was important:

…*The patient is the main priority of primary health care*. *And the more the organization has registered patients*, *the better we will live*, *because the primary health care center absolutely holds out on itself*, *as nobody pays extra* …

(woman, public primary health care, Kaunas county)

In order to keep patients, the leaders of public primary health care institutions make decisions regarding staff workload management, number of services necessary to perform, patient queue management, provision of secondary-level services to patients, employee safety, procedure and document management, and institution accreditation. The opinion that the patient should visit his/her family doctor 1–2 times a year was expressed. Public primary health care institutions solve problems related to the registration of patients with family doctors, family doctor’s visits to patient’s home, and management of repeated visits. According to the opinion of focus groups participants, additional patients’ examination at the primary level should be properly paid. The executives of public primary health care pointed out that one of the management priorities is dissemination of healthy lifestyle among patients as well as organization and implementation of health promotion programs. Because family doctors have a heavy workload, health care professionals could carry out health promotion activities for patients. The media plays an important role in shaping a positive image of primary health care organizations in the public and informing patients about health promotion programs and projects. The leaders of private primary health care institutions reported that the key priority of the primary health care organization is provision of assistance to people based on established medical norms while striving for effective functioning of the institution. The participants from the private primary health care sector stressed that 80% of the patient’s problems were solved by the family doctor; therefore, health policy makers should pay greater attention to the primary health care institution. The executives of private primary health care organizations pointed out the place of the primary health care institution, services provided to patients (quality of services, speed and time of service provision, provision of services by phone), patients’ queue management, family doctor-patient contact, and communication culture between staff and a patient as important factors. One of the management priorities is an increase in the number of registered patients in order to provide high quality health care services to patients. Attraction of additional financial resources, interinstitutional cooperation, and partnership with social partners were mentioned as management priorities by private primary health care leaders:

… *The patient is a center*, *and we are working for this*. *Private primary health care institutions are closer to the patient* …

(man, private primary health care institution, Vilnius county)

The value of the primary health care institution could be created through workload management, accessibility, application of marketing principles, management of services, management of documents, communication, quality, financial resources, health promotion and management of decision making.

### Management of human resources

Based on the opinion of the leaders of both public and private primary health care institutions, appropriate personnel are the institution’s capital and the largest investment is human capital Table 3).

**Table 3:** Categories of perspective “management of human resources”.

Public primary health care institutions		Private primary health care institutions	
Categories	Content of category	Categories	Content of category
Responsibilities	Physician’s protection: rights, duties Family physician’s functions, family doctor’s work functions (patient’s social problems) Nursing role of family health care organization	Responsibilities	Functions of family doctor Family doctor’s responsibilities are very wide A gynecologist and a surgeon in the family health care institution
Qualification	Qualified professionals Family doctor acts as a “keeper” to direct the patient to the appropriate specialist	Qualification	Competent medical consultation Patient’s problem solution Employee professionalism Patient confidence in the staff Teamwork Staff qualifications
Motivation	Employee’s motivation Employees’ penalties / praise system Employees, their security, wages, proper evaluation, operational limits	Motivation	Employee motivation to keep employees Staff motivation The largest investment in human capital
Emotional intelligence	Employee morale, readiness for work	Emotional intelligence	CEO’s personalities Recruitment and retention
Time management and burnout syndrome	Family physician job scheduling and time for patient duration Family doctors’ work organization Family doctors’ consultations per day Management of burnout syndrome	Time management and burnout syndrome	Reduction of burnout syndrome Staff workload management
Communication	Family doctor’s communication with the patient		
Selection	Replacing inefficient staff with efficient Organization’s capital is appropriate selection of employees

… *The patient is our priority followed by our employees–the main capital of the institution*. *And it is needed to talk to them and to work with them*, *to solve problems*, *conflicts*, *etc* …

(woman, public primary health care institution, Marijampolė county)

The participants of focus groups pointed out that they solved issues related to the management of burnout syndrome among employees. A family doctor acts as “a keeper to direct patients to appropriate medical specialists.” The main priorities of human resources management are family doctors’ functions, employees’ motivation, training and development of competencies, staff work management (workload, the length of visit, management of documents), the system of employees’ penalties/praise, professionalism, and patients’ confidence in staff. The executives of private primary health care institutions stressed that one of the key management priorities were staff attraction, its retention, and managers’ personality. The main aim of the family doctor’s work is to solve the patient’s problem. The leaders of both private and public primary health care institutions agreed that work norms and responsibilities of family doctors are broad; therefore, it is important to evaluate the role of personnel of nursing and other specialties (gynecologist, surgeon, psychiatrist, and pediatrician) in family medicine. The executives from public primary health care reported that one of the most important tasks of human resources management was the replacement of an inefficient employee by more efficient one. During the focus group discussions, the principles of human resources management were expressed:

…*First of all*, *for me*, *as a head of the organization*, *an employee is the most important and that he/she would feel safe at work and would know that he/she gets a salary for the work done and he/she would be properly assessed*. *And would know clearly the defined limits of his/her activities*, *rights and obligations* …

(man, public primary health care institution, Kaunas county)

In opinion of the participants, it is necessary to determine not only staff responsibilities, but also patients should be responsible for their own health and its strengthening. The leaders from public primary health care institutions reported that it was important to ensure employees’ preparedness to work with the patient, since family doctor’s communication with the patient was indicated as the main priority of primary health care institution management.

### Management of patients

The main activities of patient management cover meeting the patient expectations, improvement in satisfaction, and effective solution of problems. The participants of focus groups reported the following key management priorities related to the patient: meeting patient expectations; quality and timely satisfaction of patient’s needs; effective solution of patient’s problems; services oriented to the patient; patient satisfaction; and communication with the patient (Table 4).

**Table 4:** Categories of “management of patients” perspective.

Public primary health care institutions		Private primary health care institutions	
Categories	Content of category	Categories	Content of category
Expectations and needs	Patient satisfaction High quality and timely satisfying needs	Expectations	Patient’s expectation satisfaction
Orientation to patients	Services oriented to the patient, closer to the patient	Value proposition	Value creation for patient Standard of service for patient
Satisfaction	The patient and his/her satisfaction	Satisfaction	Patient satisfaction
Management of services	The required volume of services	Management of services	Patient demographic structure and health care services Effective solution of patient’s problems (social, medical) Patients, their attraction, and retention
		Alternatives of choice	The patient chose a specialist, rather than an health care organization
		Responsibilities	Patient’s responsibility for health Patients’ views on “medicine for free”
		Communication	Communication with the patient

The leaders of public primary health care institutions emphasized the assurance of healthcare services needed by patients as important, while based on the opinion of the executives of private primary health care institutions, it was important to attract and retain patients. The executives of private primary health care institutions are looking for ways how to increase the value for the patient created by the primary health care institution, and therefore, are implementing service standards in the activities:

…*To create value for patients*, *as competition grows in the quality of services*. *And everyone will automatically try to do the best that the patient would feel comfortable as much as possible* …

(man, private health care institution, Vilnius county)

The patient chooses a specialist, not a health care institution, and efficient human resources management is one of the most important sources of competitive advantage. The leaders of private primary health care institutions stressed the importance to evaluate the demographic structure of patients and a portfolio of primary health care services provided to them. The participants of focus group discussions expressed their view on patients’ responsibility for their own health and its strengthening, with the priority given to lifestyle medicine. There is a prevailing opinion in the society that health care services and medicine are for free; therefore, it is necessary to raise public awareness of the health care system and health care services provided.

### Management of health policy decision-making

During focus group discussions, there was an opinion that a monopoly exists in the Lithuanian primary health care market, and increasing competition would bring positive effects such as improvement in health care quality and an increase in patient satisfaction:

… *Sincere communication with the patient and constant his/her acceptance*, *not rejection*, *are very important in competition* … (woman, public primary health care institution, Utenos county)

Unequal conditions of competition reduces the ability to compete and to provide health care services more efficiently. The content of “Health Policy Decision-Making” category are presented in Table 5.

**Table 5:** Categories of “health policy decision making” perspective.

Public primary health care institutions		Private primary health care institutions	
Categories	Content of category	Categories	Content of category
Management of services	Accessibility of services Geographical accessibility Electronic means Quality of services and volume of the services	Management of services	Geographical accessibility Quality Electronic medical history
Partnership	Cooperation between primary and secondary health care levels	Partnership	Cooperation between organizations
Strategy	Health policy and strategy of the primary health care organization Health system structure and management (patient flows between the primary and secondary level, the differences between urban and rural regions) The priority of secondary level to perform examination at the primary level Fever services by doctors specialist consultants	Strategy	Philosophy of primary medicine Family doctors patients’ parish sizes Concept of family physician specialty Communication between a family doctor and a specialist consultant Cooperation between primary and secondary levels ensuring an effective health care reform Communication between the primary and secondary levels Teamwork at the primary health care level
Competition	Competition is based on cash flow management The absence of competition does not ensure the competitiveness of service contracts Unequal conditions of competition in a regional approach	Competition	Monopoly Larger health care institutions have greater opportunities to compete than smaller medical organizations Priority is given to large health care organizations, rather than a family doctor’s organization Allocation of financial resources
Gaps management	Management of Ministry of Health institution, communication with the first-level employees, allocation of funds, lack of diseases treatment options Communication among primary, secondary, tertiary levels Lack of family doctors Reorganization of medical centers	Gaps management	Weaknesses of services payment Search for funding resources with a focus on innovation Reorganization of medical centers Social services
Legal regulation	Legal framework regulating the work of public health care organizations Legal regulations of doctors’ work Political decisions	Legal regulation	Compliance of legal regulation with reality
Health promotion	Disease prevention, patient health strengthening Functions of public health offices		
Model	Mixed model (teamwork at primary health care level) Incentive services in primary health care organization, fees of services, incentive programs Management of family physician payment rates Social services in primary health care organization		
Society	Public involvement in the formation of healthy lifestyle and patient flow formation Demographic changes (population ageing)		

Competition is based on the management of financial flows, and the absence of competition does not guarantee competitive procurement and provision of services. The participants of focus group discussions reported the following major gaps in the health care market: health policy and strategy, structure and management of the health system (patient flows among primary, secondary, and tertiary levels; inequalities between rural and urban areas), concept of family physician specialty, assurance of teamwork at the primary health care level, reorganization of medical institutions, lack of substantiation of philosophy of a family medicine institution, cooperation with public health offices, allocation of funds, lack of treatment options, involvement of the public in the formation and dissemination of healthy lifestyle, legal regulation and compliance with real family medicine work, shortage of family doctors, and attraction of human resources to rural areas. The executives of both public and private primary health care institutions pointed out that the key management priorities were related to accessibility to services, geographical accessibility, quality, disease prevention, and patients’ health strengthening:

…*I think that work management at first*, *then safety and its requirements followed by human resources and the quality of services* …

(woman, public primary health care institution, Klaipėda county)

This study of focus groups showed that the management priorities of primary health care institutions include the management of an organization, human resources management, patient management, and health policy decision-making. Setting the priorities in the management of the institution is essential and depends on ownership of the institution and leadership of management.

## Discussion

Competitiveness among primary health care institutions develops an exclusive position in the market, and value creation for patients provides a competitive advantage and helps the health care institution to achieve exceptional positions in the health care sector [19]. Improvement in the management of primary health care institutions increases competitiveness; therefore, setting the priorities of value creation and selection of competitive abilities are essential both for the patient and the health care system. The main priorities of service management are human resources management, value creation, management of changes [20], as well as orientation to a customer and expertise [21]. Other studies pointed out that the strategic priority of an organization was to achieve long-term satisfaction of customers [22]. In the UK, patient-centered care and patient involvement are the central priorities of health policy [23]. In Thailand, competitive priorities for services providers were examined, and quality (34.5%), service provision (20.4%), orientation to customer (12.9%), and know-how (12.5%) were found to be the most important priorities [24]. The qualitative study carried out by Pinho et al. in Portugal explored the factors of value co-creation and showed that a holistic view to a patient, available information, communication, and information availability to a patient were the main elements of value co-creation [25]. In the US, the essential priorities of the health care system are quality of well-being and cost effectiveness [26]. In Norway, the following priorities of the health care system were acknowledged: severity of condition, effect, and cost-effectiveness [27]. With the help of a Grounded theory approach, communication, strategy of management, and partnership were founded to be priorities in public health [28]. In the US, the priorities of competitiveness in public hospitals are quality, price, production, flexibility, and performance [29]. Hospitals create trust with patients and the community and evaluate the performance by clinical outcomes, patient satisfaction, and financial results [30]. In the ageing society, the priority of primary health care institutions is chronic disease management in line with policy initiatives such as changing care processes, developing relationships with society, maintaining patient management, using clinical information systems, and teamwork building with community nurses [31]. While managing chronic diseases in the primary health care institution, the staff recognizes the fundamentals values of practice: quality, ethics, and orientation to patients [32]. Scientific literature discusses about the determination of health care priorities based on social values such as transparency, accountability, participation, clinical effectiveness, cost-effectiveness, equity/justice, solidarity, and autonomy [33]. Previous studies conducted in Lithuania investigated the priorities of value creation for patients and competitive abilities of health care services: patient satisfaction [34]; patient expectations [35]; patients’ role in the decision-making process [36, 37]; accessibility to health care services [38, 39]; quality of health care services [40–42]; patients’ confidence in health care services [43]; importance of modern health care management [44]; stress management in family doctors’ work [45]; nurses’ satisfaction with job [46–48]; and application of marketing elements in health care institutions [49]. The significance of the qualitative study is based on theoretical and practical importance. The theoretical significance is evident because the scientific issue on priorities in the management of primary health care has received little attention by carrying out qualitative studies. The study results can be root for arising of scientific management theories. The practical importance of the study is related to the results of focus group research and its can be a strategic directions for developing the health care reform in Lithuania. Focus groups discussions revealed that the priorities in the management of primary health care institutions included work organization, human resources management, management of patients, and health policy decision making. In our study, the executives of public health care institutions stressed expectations and needs, orientation to patients, satisfaction, and management of services to be the main priorities of patient’ management; while the leaders of private health care institutions reported that the priorities of patient management were expectations, value proposition, alternatives of choice, management of services, satisfaction, responsibilities, and communication. Scientific literature discusses about human resources as a key source of competitive advantage of the institution. The findings of focus group study showed that priorities in the management of human resources included responsibilities, qualification, motivation, selection, time management and management of burnout syndrome, communication and emotional intelligence. The participants of focus group sessions paid attention to employee’s responsibilities and motivation. The executives from private primary health care institutions paid attention to marketing principles in work management of the institution, while the leaders from public primary health care institutions considered image-building of the institution as important. Value creation for patients is based on the mechanism of values and results for patients including effective meeting the patients’ expectations and needs, health improvement, accessibility, satisfaction, and quality.

## Conclusions

1. Effective work organization of primary health care services helps to achieve a competitive advantage in the market.
2. Motivated and qualified staff of the primary health care institution is the main capital and element of competitive advantage.
3. Meeting the patient expectations, patient satisfaction, and effective problem solving are essential solutions of patient management.
4. Sources of competitiveness in primary health care institutions include a unified e-system throughout the country, improvement in the quality of services, improvement in tariff management of services provided by the family doctor, provision of social services, and search for funding resources with an emphasis on innovations.

### Study limitations and directions for future research

In any scientific research, there are a number of study limitations and future directions. The strengths of this focus group study carried out in Lithuania could be mentioned: the executives of both public and private primary health care institutions were invited to participate in this study and the study involved the participants from all counties of the country. One of the limitations of this study is that focus group discussions involved the executives from only primary health care level institutions, and future research should enroll participants from secondary and tertiary levels. The researchers expressed that the study involved the managers from primary health care institutions and it would be interesting to research the opinion of patients about priorities of primary care. The researchers point out that this study can be the direction for future research in order to compare the results with the findings in other countries. In management science this qualitative study and the results can be a basis for the health care reform development and the foundation for new theories.
